# Palliativmedizin und Sterben im Rettungsdienst – Einstellungen, Sichtweisen und Kenntnisse von Notärzten

**DOI:** 10.1007/s00063-025-01313-5

**Published:** 2025-08-25

**Authors:** Susanne Glass, Magdalena Bier, Celina Pötz, Lukas Jäger, Tobias Grübl, Pia von Blanckenburg, Christian Volberg

**Affiliations:** 1 Akut- und Notfallmedizin, Diakonie-Krankenhaus Wehrda, Marburg, Deutschland; 2 Klinik für Anästhesie und Intensivtherapie, Universitätsklinikum Marburg, Philipps-Universität Marburg, Baldingerstraße, 35043 Marburg, Deutschland; 3 Klinik X – Anästhesie, Intensivmedizin, Notfallmedizin und Schmerztherapie, Bundeswehrzentralkrankenhaus Koblenz, Koblenz, Deutschland; 4 Institut für klinische Psychologie und Psychotherapie, Fachbereich Psychologie, Philipps-Universität Marburg, Marburg, Deutschland

**Keywords:** Palliativversorgung, Notfallmedizin, Lebensende, Versorgung, Ausbildung, Palliative medicine, Emergency medicine, End-of-life care, Support, Education

## Abstract

**Hintergrund:**

Notärzte werden im Einsatzdienst fast täglich mit dem Thema Tod und Sterben konfrontiert. Demografischer Wandel und die damit einhergehende Zunahme an chronischen sowie nichtheilbaren Erkrankungen am Lebensende, aber auch der immer häufiger geäußerte Wunsch im häuslichen Umfeld zu sterben, führen zu einer Zunahme palliativer Akutsituationen. Genaue Daten zum Umgang mit palliativen Patienten im Notarztdienst liegen bis heute nur unzureichend vor.

**Methode:**

Notärzte zweier Leistungserbringer konnten an der Befragung „Palliativmedizin und Sterben im Rettungsdienst“ teilnehmen. In einer Paper-Pencil-Befragung wurden die Notärzte über mehrere Themenkomplexe in den Bereichen Palliativmedizin, Beschreibung von Konflikten bei nicht originär notfallmedizinischen Einsätzen sowie der persönlichen Einstellung zu Sterben und Tod in Anlehnung an bereits etablierte psychometrische Standards befragt. Die Erhebung erfolgte anonymisiert.

**Ergebnisse:**

Die Rücklaufquote betrug 40 % (*n* = 67). Das durchschnittliche Alter der Teilnehmer betrug 41 Jahre, 66 % (*n* = 44) waren männlich. Die deutliche Mehrheit der befragten Notärzte waren Anästhesisten und bereits über 5 Jahre als Notarzt tätig. Nur sechs Befragte (9 %) gaben an, eine Zusatzweiterbildung in Palliativmedizin zu haben. Im Umgang mit Sterbenden fühlten sich die berufserfahreneren Ärzte im Durchschnitt besser vorbereitet als die Kollegen, die weniger als 5 Jahre Berufserfahrung als Notarzt hatten. Die Erhebung zeigt auf, dass bei vielen Notärzten ein Konflikt zwischen Sterbebegleitung und Leben retten zu existieren scheint, wenn Palliativpatienten behandelt werden. Keiner der Befragten gab an, dass Palliativmedizin nichts mit Notfallmedizin zu tun habe. Die befragten Notärzte fühlen sich unsicher bei der akuten präklinischen Versorgung von Palliativpatienten.

**Schlussfolgerung:**

Die Kompetenz in Palliativmedizin steigt bei Notärzten mit der Einsatzerfahrung. Nahezu alle befragten Notärzte empfinden diesen Themenkomplex als sehr wichtig und wünschen sich eine stärkere Integration der Inhalte in die Aus- und Fortbildung. Aus der Unsicherheit heraus besteht die Möglichkeit, dass Palliativpatienten weniger sinnhafte Therapien angeboten werden, die z. B. in einer Klinikeinweisung enden. Der demografische Wandel wird den Bedarf an palliativmedizinischer Kompetenz bei Notärzten steigern. Die aufgezeigte Diskrepanz gilt es zu beheben.

## Abstract (en)

**Background:**

Emergency physicians are confronted with the topic of death and dying almost daily in their work. Demographic changes and the accompanying increase in chronic and incurable diseases at the end of life as well as the frequently expressed wish to die at home, are leading to an increase in emergency situations with palliative patients. To date, there is insufficient data available on how emergency physicians deal with palliative patients.

**Method:**

Emergency physicians from two service providers participated in the survey ‘Palliative Care and Dying in Emergency Medical Service’. The emergency physicians were asked to complete a paper-and-pencil survey covering several topics in the areas of palliative medicine and descriptions of conflicts in non-emergency medical situations. They were also asked about their personal attitudes towards dying and death, based on established psychometric standards. The survey was conducted anonymously.

**Results:**

The response rate was 40% (*n* = 67). Participants had an average age of 41 years, and 66% (*n* = 44) were male. The majority of emergency physicians surveyed were anaesthetists and had worked as emergency physicians for more than five years. Only six respondents (9%) had an additional training in palliative medicine. On average, more experienced doctors felt better prepared to deal with dying patients than those with less than five years’ professional experience as emergency physicians. The survey showed that many emergency physicians experience a conflict between end-of-life care and saving lives when treating palliative patients. None of the respondents stated that palliative medicine had nothing to do with emergency medicine. The emergency physicians surveyed expressed uncertainty regarding the acute preclinical care of palliative patients.

**Conclusion:**

The expertise of emergency physicians in palliative medicine increases with the level of operational experience. Almost all respondents consider this topic to be very important and would like to see it integrated more strongly into training and continuing professional education. A lack of knowledge may result in palliative patients being offered less meaningful therapies, which could lead to hospitalisation. Demographic changes will increase the need for emergency physicians with expertise in palliative medicine. The discrepancy identified must be addressed.

Sterben und Tod sollten von Mitarbeitenden der Gesundheitsberufe als etwas Natürliches betrachtet werden, das zum Berufsbild dazu gehört. Im Rahmen der präklinischen Versorgung wird das Rettungsdienstpersonal in diesem Zusammenhang jedoch nicht nur mit medizinischen, sondern auch mit ethisch-moralischen Fragestellungen konfrontiert. Oftmals zeigt sich hierbei ein Spannungsfeld [4, 13]. Die primäre Aufgabe des Rettungsdienstes in der Notfallmedizin sieht zumeist einen kurativen Behandlungsansatz mit Stabilisierung der Vitalparameter, lebensrettenden Interventionen und letztlich dem Abwenden von potenzieller Lebensgefahr mit Versterben oder schweren Folgeschäden vor. In der Palliativmedizin hingegen ist die begleitende Behandlung auf möglichst geringen Einsatz von Diagnostik und invasiver Therapie ausgelegt [14]. Die Lebensqualität der Patienten soll verbessert werden, ohne eine Lebenszeitverlängerung um jeden Preis zu erzielen [15]. Die Autonomie und die individuellen Vorstellungen des Einzelnen werden hierbei als wichtigste Säule in der Behandlung angesehen und das Versterben als natürlicher Prozess des Lebens betrachtet [1]. Somit stellen Einsätze bei Palliativpatienten eine besondere Herausforderung dar, da hier Notfall- und Palliativmedizin auf einen gemeinsamen Nenner für den Patienten zu bringen sind und die besondere Situation von den Rettungsdienstmitarbeitern innerhalb kürzester Zeit entsprechend erkannt werden muss. Die Gründe einer notfallmäßigen Konsultation sind mannigfaltig und reichen von Symptomexazerbationen, akuten Erkrankungen unabhängig von der palliativen Grunderkrankung (z. B. ACS) und Nebenwirkungen von Therapien, neuaufgetretenen Problemen im Zusammenhang mit der Grunderkrankung (z. B. Frakturen bei Knochenmetastasen) über Ganzkörperschmerz bis zur Überforderung der An- und Zugehörigen in der Sterbephase der Patienten [5]. Es existieren verschiedene Tools, wie beispielsweise die „Mainzer Checkliste Palliativpatienten in der Notfallmedizin“, welche Krankenhauseinweisungen reduzieren kann [1]. Leider fehlen einheitliche Standard Operation Procedures (SOPs) und somit obliegt es den Ärzten oder Notfallsanitätern an der Einsatzstelle, welche Therapie in der Akutsituation durchgeführt wird und ob eine Behandlung im Krankenhaus notwendig ist [1]. In der Versorgung der Patienten sollen medizinethische Aspekte idealerweise nach der Prinzipienethik von Beauchamp und Childress berücksichtigt werden [22]. Hierbei handelt es sich um: Autonomie des Patienten, Schadensvermeidung, Fürsorge und Gerechtigkeit. Dabei stellt die Alltagsmoral den Ausgangspunkt für die Prinzipienethik dar. Ebendiese soll in den Prozess der ethischen Begründung und letztlich der Entscheidungsfindung mit einbezogen werden [21]. Etwaige ethisch-juristische Fragestellungen, beispielsweise die Aktualität und Gültigkeit einer vorgelegten Patientenverfügung, aber auch die Sinnhaftigkeit einer Akutbehandlung und die Frage nach dem Therapieziel, führen häufig zu Belastungen des Einzelnen oder aber auch innerhalb des Teams [2]. Van Oorschot et al. konnten 2005 in einer Befragung von insgesamt 727 Ärzten (hier Internisten, Anästhesisten und Allgemeinmediziner) herausarbeiten, dass die große Mehrheit eine vorliegende Patientenverfügung bei einer Entscheidungsfindung als relevant erachtet und diese als Kommunikationsinstrument dient [23]. Darüber hinaus können vorliegende Patientenverfügungen als eine Entlastung für die Angehörigen dienen. Weiterhin konnte gezeigt werden, dass Patientenverfügungen bei den befragten Ärzten allgemein eine hohe Wertschätzung erfahren, jedoch eine Skepsis gegenüber einer stellvertretenden Entscheidung von Betreuern und/oder Bevollmächtigten existiert [23]. Im Gegensatz dazu erörtern Roessler und Eulitz in ihrer Studie von 2018, dass die Frage nach einer vorhandenen Patientenverfügung im Rahmen einer präklinischen Versorgung bei den Behandelnden zu einer „Aussichtslosigkeitsannahme“ führen kann bzw. von Angehörigen dahingehend missverstanden werden kann, dass grundsätzlich keine Maßnahmen mehr getroffen werden sollen und sodann auch beim Notarzt eine „negative therapeutische Grundeinstellung“ entsteht [4]. Roessler und Eulitz bestätigten auch, dass eine palliativmedizinische Situation in der Präklinik sowohl eine medizinische als auch eine psychosoziale Herausforderung für den gerufenen Notarzt vor Ort darstellt [4]. Liegt keine Patientenverfügung vor, kann im Fall, dass der Patient selbst keine medizinischen Entscheidungen mehr treffen kann, die medizinische Behandlung auch durch dritte Personen wie Angehörigen, Betreuer oder Bevollmächtigte mitbestimmt werden. Dies kann zu einer Behandlung führen, die nicht dem mutmaßlichen Willen des Patienten entspricht. Die Auswirkungen des Fehlens oder der Nichtgültigkeit einer Patientenverfügung auf eine konkrete Situation sind dabei mannigfaltig. Dabei ist die Kommunikation am Lebensende für Patienten und deren Angehörige mit einer lebenslimitierenden Erkrankung in der Palliativmedizin „normal“ und stellt die Basis in einer guten Arzt-Patienten-Beziehung dar [24]. Hierfür hat man in der Palliativversorgung verhältnismäßig mehr Zeit für ausführliche Gespräche, und darüber hinaus liegen zumeist alle relevanten Befunde vor. Die Kommunikation am Lebensende in der Akutsituation unterliegt jedoch anderen Voraussetzungen und somit einer anderen Ausgangssituation, sowohl medizinische Informationen, Prognose und Behandlungsverlauf betreffend, als auch einer anderen Arzt-Patienten-Beziehung. Hier wird eine Ad-hoc-Kommunikation mit weniger Vorbereitung in Bezug auf (Vor‑)Informationen und zeitkritische Entscheidungen verlangt. Die Kommunikation im Notarztdienst mit Palliativpatienten und deren Angehörigen beruht somit auf der Berücksichtigung der kommunikativen Grundregeln und weniger auf Kommunikationsmodellen wie beispielsweise dem „SPIKES-Protokoll“ [24].

Bereits 2010 konnte durch Ruppert dokumentiert werden, dass sowohl Notärzte als auch nichtärztliches Rettungsdienstpersonal über keine ausreichende Kenntnis zu den Themenkomplexen Palliativmedizin und Umgang mit Patientenverfügung in entsprechenden Situationen verfügten. Hier hatten weniger als ein Zehntel der Befragten etwas über Palliativmedizin gehört, jedoch mehr als 90 % bereits Einsätze mit palliativmedizinischen Fragestellungen erlebt. Es konnte der Wunsch nach palliativmedizinischer Aus‑, Fort- und Weiterbildung abgeleitet werden [3]. Ob sich an dem von Ruppert beschriebenen Sachverhalt seitdem etwas geändert hat, ist unklar, so dass die vorliegende Erhebung diesbezüglich Erkenntnisse bringen soll.

## Hintergrund und Fragestellung

In dieser Querschnittsstudie wurde der Kenntnis- und Wissensstand von Notärzten in zwei verschiedenen Rettungsdienstbereichen über den Themenkomplex Palliativmedizin, der Beschreibung von Konflikten bei nicht originär notfallmedizinischen Einsätzen sowie die persönliche Haltung zu Sterben und Tod untersucht. Unter nicht originär notfallmedizinischen Einsätzen werden Notfalleinsätze verstanden, welche nicht das Ziel einer lebensrettenden Intervention im kurativen Sinne innehaben, sondern vielmehr einen sozialmedizinischen oder palliativmedizinischen Aspekt (z. B. Organisation der Versorgung oder Sterbebegleitung) verfolgen. Im Vordergrund der Erhebung standen hierbei folgende Fragen:

1. Wie ist die Einstellung von Notärzten zu Tod und Sterben? Hat die tägliche Konfrontation mit schwerstkranken und sterbenden Menschen einen Einfluss auf das eigene Leben? Zum Beispiel, ob Notärzte vermehrt Vorsorgevorkehrungen im Sinne einer eigenen Patientenverfügung im Vergleich zur Allgemeinbevölkerung erstellen.
2. Welchen Kenntnisstand haben Notärzte über Palliativmedizin, damit einhergehende Krankheitsbilder, Therapieoptionen und Möglichkeiten der Versorgung von Palliativpatienten?
3. Existieren Konflikte zwischen Leben retten und Sterbebegleitung, wenn im Einsatzgeschehen zum Beispiel Patienten im Finalstadium einer zum Tode führenden Erkrankung behandelt werden müssen oder hochbetagte Patienten reanimiert werden?

## Material und Methodik

Die multizentrische Querschnittsstudie wurde als ein Gemeinschaftsprojekt der Arbeitsgruppe Ethik in der Medizin am Fachbereich Humanmedizin der Philipps-Universität Marburg, dem Zentrum für Notfallmedizin am Universitätsklinikum Marburg, dem Institut für klinische Psychologie und Psychotherapie der Philipps-Universität Marburg sowie den beiden Leistungserbringern DRK Rettungsdienst Mittelhessen gGmbH und dem Notarztstandort am Bundeswehrzentralkrankenhaus in Koblenz durchgeführt.

Die Fragebögen wurden nach ausführlicher Literaturrecherche zu der Thematik durch das Studienteam neu entwickelt. Um eine bessere Vergleichbarkeit zu erzielen, wurden bereits validierte Fragebögen in den Fragenkatalog integriert. Persönliche Einstellungen und Erfahrungen der Teilnehmer wurden anhand einer fünfstufigen Likert-Skala abgefragt. Vor dem Start der Befragung wurden die Fragebögen von fünf ärztlichen Kollegen auf Verständlichkeit überprüft und im Nachgang in kleinen Punkten überarbeitet und angepasst.

Die Befragung fand als Paper-Pencil-Befragung statt. Die zurückgesendeten Fragebögen wurden in die IBM® SPSS® Statistics Software Version 21.0 (IBM, Armonk, NY, USA) übertragen und ausgewertet.

Es erfolgte ein deskriptives Auswertungsverfahren, mit Berechnung der deskriptiven Maße sowie der zugehörigen Konfidenzintervalle. Für nichtnormalverteilte Variablen wurden die entsprechenden nichtparametrischen Lagemaße berechnet (Median, Interquartilsabstand). Unterschiede zwischen den Notärzten, wie beispielsweise die Fachrichtung, die Arbeitsjahre etc. wurden mithilfe von multivariaten Varianzanalysen festgestellt. Mögliche Zusammenhänge zwischen unterschiedlich erhobenen Variablen wurden mittels zweiseitigem t‑Test analysiert. Das Signifikanzniveau wurde auf *p* < 0,05 festgelegt.

## Studiendesign und Untersuchungsmethoden

Nach positivem Ethikvotum der zuständigen Ethikkommission der Philipps-Universität Marburg am 11.03.2021 (AZ 41/21) wurden im Zeitraum zwischen März und August 2021 die Fragebögen verteilt. Am 09.11.2022 erfolgte das positive Zweitvotum der Ethikkommission der Landesärztekammer Rheinland-Pfalz (AZ 2022-16742). Die Erhebung am Bundeswehrzentralkrankenhaus in Koblenz erfolgte zwischen Dezember 2022 und März 2023. Die Studie wurde im Deutschen Register Klinischer Studien (DRKS00026194) registriert. Insgesamt 168 Notärztinnen und Notärzte zweier Leistungserbringer hatten die Möglichkeit, an der Erhebung auf freiwilliger Basis teilzunehmen. Die Erhebung erfolgte anonymisiert, indem auf den entsprechenden Notarztwachen die Fragebögen in Papierversion zum Ausfüllen bereitlagen. Der Fragebogen umfasste verschiedene Bereiche aus validierten Instrumenten. Die selbstformulierten Fragen waren ein Konsens des Teams. Erfasst wurden demografische Daten der Teilnehmenden, wie Fachgebiet, vorhandene Zusatzbezeichnungen, Diensterfahrung in Jahren, Konfession sowie einige Items aus den validierten Fragebögen PCEP-GR (Palliative Care Education and Practice Questionnaire – German Revised Version), FIMEST‑E (Fragebogeninventar zur mehrdimensionalen Erfassung des Erlebens gegenüber Sterben und Tod), PHQ‑2 (Patient Health Questionnaire-2), GAD‑2 (Generalized Anxiety Disorder Scale-2), BPW (Bonner Palliativwissentest) sowie ein Freitext-Teil (vgl. Tab. 1).

**Tab. 1:** Darstellung der psychometrischen Fragebögen

*PCEP-GR* (Palliative Care Education and Practice Questionnaire – German Revised)	Dieser Fragebogen ist die deutsche Version des „Palliative Care Education and Practice“ und erfasst die Erfahrung und den persönlichen Umgang mit Sterbenden
*FIMEST‑E* (Fragebogeninventar zur mehrdimensionalen Erfassung des Erlebens gegenüber Sterben und Tod)	Dieser Fragebogen erfasst und differenziert die emotionale Bewertung einer Person während der gedanklichen Beschäftigung mit Sterben und Tod. Hierzu werden 8 Subtests und 47 Items genutzt
*PHQ‑2* (Patient Health Questionnaire-2)	Dieser Test besteht aus 2 Fragen für ein Screening einer Major-Depression nach DSM IV und stellt die Kurzversion des PHQ‑9, eines vollständigen Depressionsmoduls des PHQ‑D, dar. Hierbei werden die Ausprägungen beider Fragen addiert, ein Summenwert von 0 bis 6 ist möglich, der Trennwert (Cut-off-Wert) von ≥ 3 für das Screening wird als optimal beschrieben
*GAD‑2* (Generalized Anxiety Disorder Scale 2)	Dieser Test ist ein kurzes Screening-Instrument zur Erfassung von generalisierten Angststörungen, welcher aus dem GAD‑7 hervorgegangen ist. Zudem etablierte sich dieser Fragebogen aus dem Fragenpool für Patienten (PHQ-D). Hier wird eine 4‑stufige Skala genutzt
*BPW* (Bonner Palliativwissenstest)	Dieser Test dient der Erfassung von Wissen über Palliativversorgung, hier gibt es 23 Items und für den Selbstwirksamkeitstest 15 Items

Als Einschlusskriterium galt die Tätigkeit als Notarzt in einem der beiden Rettungsdienstbereiche. Für die Auswertung wurden alle zurückerhaltenen Fragebögen eingeschlossen. Wurden von den Befragten einzelne Fragen nicht beantwortet, wurden diese Fragen nicht ausgewertet, ohne den gesamten Fragebogen auszuschließen. Als Ausschlusskriterium galt die fehlende Bereitschaft, an der Erhebung teilzunehmen.

## Ergebnisse

Im Erhebungszeitraum ergab sich eine Gesamt-Rücklaufquote von 40 % (*n* = 67). Eine Übersicht der demografischen Charakteristika der Studienteilnehmenden zeigt Tab. 2.

**Tab. 2:** Demografische Daten der Teilnehmenden

	Gesamtkollektiv
Alter in Jahren (MW ± SD)	40,7 ± 9,2
Geschlechterverteilung	
Männlich	44 (65,7 %)
Weiblich	23 (34,3 %)
Fachgebiet	
Anästhesie	49 (73,1 %)
Innere	6 (9 %)
Chirurgie	5 (7,5 %)
Sonstige	7 (10,4 %)
Zusatzbezeichnung	
Palliativmedizin	6 (9 %)
Intensivmedizin	27 (40,3 %)
Aktive Zeit als Notarzt/Notärztin	
< 5 Jahre	26 (38,8 %)
5–10 Jahre	10 (14,9 %)
10–15 Jahre	15 (22,4 %)
> 15 Jahre	16 (23,9 %)
NA-Dienste pro Jahr	
< 10	8 (11,9 %)
10–50	39 (58,2 %)
50–100	15 (22,4 %)
> 100	5 (7,5 %)
Vorhandensein einer eigenen Patientenverfügung	
Ja	44 (65,7 %)
Nein	23 (34,3 %)

Bezugnehmend auf die erste Fragestellung zur persönlichen Einstellung zu Sterben und Tod, konnte gezeigt werden, dass in der befragten Kohorte 65,7 % (*n* = 44) eine eigene Patientenverfügung besitzen, als Zeichen der aktiven persönlichen Auseinandersetzung mit dem Thema Versorgung am Lebensende. Mittels PCEP-GR wurde untersucht, ob die befragten Notärztinnen und Notärzte ausreichend auf die Versorgung Sterbender vorbereitet sind. Die Befragten fühlten sich ausreichend vorbereitet (*Median* *=* *3,85; SD* *=* *0,95*). Dies wurde zwischen Gesamtwert des PCEP-GR und der aktiven Zeit im Rettungsdienst und/oder als tätiger Arzt bestätigt. Mit Zunahme der Tätigkeit in der präklinischen Versorgung zeigte sich eine signifikant bessere Vorbereitung auf diese Situationen (*p* *=* *0,036*). Im Subgruppenvergleich Berufserfahrung ≤ 5 Jahre und > 10 Jahre zeigte sich, dass diejenigen Kollegen mit mehr Berufserfahrung sich sicherer im Umgang mit klassischen palliativmedizinischen Fragestellungen, wie behandeln von Dyspnoe, Bedürfnisse der Palliativpatienten erkennen sowie Sterbebegleitung fühlen. Eine vollständige Übersicht findet sich in Tab. 3.

**Tab. 3:** Fragen mit signifikanten Unterschieden im Subgruppenvergleich, basierend auf der Berufserfahrung

Frage	≤ 5 Jahre Berufserfahrung		> 10 Jahre Berufserfahrung		*p*	95 % KI
	*n*	MW ± SD	*n*	MW ± SD		
Sich vorbereitet fühlen, um Sterbende zu begleiten	26	3,62 ± 1,023	30	4,13 ± 0,776	0,036	[−1,001, −0,035]
Sich vorbereitet fühlen, um Dyspnoe am Lebensende zu behandeln	26	4,00 ± 0,748	30	4,47 ± 0681	0,018	[−0,850, −0,084]
Sich vorbereitet fühlen, um mit den emotionalen Bedürfnissen des Patienten umzugehen	26	3,65 ± 0,892	30	4,17 ± 0,747	0,023	[−0,952, −0,074]
Sich vorbereitet fühlen, um mit religiösen Themen des Patienten umzugehen	26	2,85 ± 0,925	30	3,43 ± 0,898	0,02	[−1,076, −0,098]
Mehr Fortbildung zu Palliativmedizin gewünscht	26	4,00 ± 0,748	31	3,39 ± 0,989	0,012	[0,140, 1,086]
Es gibt Situationen, da fühle ich mich im Zwiespalt zwischen Notfall- und Palliativmedizin	26	3,92 ± 0,845	31	3,26 ± 1,264	0,026	[0,082, 1,248]
Ich weiß welche Aufgabe das Palliative-Care-Team übernimmt	26	2,88 ± 0,909	31	3,52 ± 1,061	0,02	[−1,162, −0,102]
Wunsch nach Algorithmus zum Umgang mit Sterbenden	25	2,92 ± 0,997	31	2,35 ± 1,050	0,045	[0,012, 1,119]
Mich erschreckt der Gedanke, dass mit dem Tod mein gesamtes Denken und Fühlen aufhört	26	0,96 ± 0,916	31	0,45 ± 0,768	0,026	[0,063, 0,957]
Die Vorstellung, dass mit meinem Tod meine ganze Persönlichkeit verschwindet, ist entsetzlich für mich	26	0,77 ± 0,587	31	0,39 ± 0761	0,041	[0,016, 0,748]
Die Vorstellung, dass ich nach meinem Tod nie wieder denken und Erfahrungen machen kann, beunruhigt mich	26	0,81 ± 0,694	31	0,29 ± 0,643	0,005	[0,162, 0,873]
Mein Tod ist Teil einer umfassenderen Ordnung, die ich bejahe	26	1,77 ± 0,909	31	2,32 ± 0,702	0,012	[−0,981, −0,126]
Wie viele Reanimationen pro Jahr	26	9,23 ± 6,843	30	14,90 ± 8,327	0,008	[−9,792, −1,546]
FIMEST_Angst_Tod	14	0,845 ± 0,372	21	0,492 ± 0,515	0,034	[0,027, 0,679]

In Bezug auf die Frage nach dem Kenntnisstand zu palliativmedizinischen Krankheitsbildern, Therapieoptionen und Versorgung von Palliativpatienten, wurde mithilfe des BPW das Antwortverhalten der Befragten untersucht. Die Notärzte konnten die richtigen und falschen Aussagen erkennen (*Mittelwert* *=* *2,94; SD* *=* *0,47*). Der Wert von „3“ im BPW der Antwortmöglichkeiten bedeutet hier, „stimmt eher“. Nur der Wert „4“ („stimmt“) zeigt eine noch deutlichere Zustimmung zur jeweiligen Aussage. Ausgehend von diesen Ergebnissen lässt sich zusammenfassen, dass ein guter Kenntnisstand der Befragten vorlag. Eine mögliche Einflussnahme durch die Zusatzweiterbildung Palliativmedizin konnte nicht aufgezeigt werden, da nur 9 % (*n* = 6) der Teilnehmer über die Zusatzweiterbildung Palliativmedizin verfügten.

Frage 9 („Es gibt Situationen, da fühle ich mich im Zweispalt zwischen Notfall- und Palliativmedizin“) des selbstkonfigurierten Fragebogens zielte auf Konflikte in Bezug auf die Versorgung von Patienten im Finalstadium einer zum Tode führenden Erkrankung ab. Die Antworten ergaben einen Mittelwert von 3,54 ± 1,09 auf der Fünfer-Likert-Skala. Ableitend ist hier zusammenzufassen, dass die Befragten in gewissen Situationen sich in einem Zwiespalt zwischen den zwei Tätigkeitsfeldern befinden. Hier konnte gezeigt werden, dass die erfahrenen Kollegen und auch die Kollegen mit Zusatzweiterbildung weniger Schwierigkeiten haben. Zudem sollte die Frage nach „Notfallmedizin hat nichts mit Palliativmedizin zu tun“ (Frage 14) Aufschluss über die Wahrnehmung eines möglichen Zwiespalts geben. Der Mittelwert von 1,52 ± 0,77, lässt den Rückschluss zu, dass die Befragten hier der Ansicht waren, dass Notfallmedizin häufig Aspekte der Palliativmedizin enthält. Die Frage zum Wunsch nach einem Algorithmus als Handlungsgrundlage zum Umgang mit Sterbenden (Frage 24) sollte klären, ob sich hier ein aktiver Handlungsbedarf ergibt. Es zeigte sich ein Mittelwert von 2,61 ± 1,01, was einer ambivalenten Einstellung zu dem Wunsch eines möglichen Algorithmus entspricht. Die Frage nach einem Wunsch zu mehr Fortbildung im Bereich Palliativmedizin beantworteten die Teilnehmenden mit einem Mittelwert von 3,61 ± 0,92. Einen Wert von 4,11 ± 0,77 ergaben die Antworten zur Frage, ob Palliativmedizin Bestandteil der Aus- und Weiterbildung sein sollte.

## Diskussion

In mehreren Studien, unter anderem von Wiese et al. aus 2008 und von Roessler und Kollegen aus 2018 konnte belegt werden, dass palliativmedizinische Einsätze im Rettungsdienst heutzutage keine Seltenheit mehr sind [4, 5]. Michels und Kollegen bestätigen in ihrem Konsensuspapier von 2023, dass, basierend auf der derzeitigen demografischen Entwicklung und der damit implizierten Zunahme von chronischen Erkrankungen in unserer Gesellschaft, die Notarzteinsätze bei Palliativpatienten bereits einen Anteil von 10 % einnehmen [6]. In unserer Studie konnten wir exemplarisch für zwei Rettungsdienstbereiche zeigen, dass in der Kohorte der Befragten zufriedenstellende Grundkenntnisse über die Themenkomplexe rund um die Palliativmedizin existieren. Anhand einiger ausgewählter Fragen konnte der Rückschluss gezogen werden, dass mit zunehmender Berufserfahrung die Routine im Umgang mit Palliativpatienten in der Präklinik größer wird. Jenseits der zehn Jahre Berufserfahrung und dem damit verbundenen Facharztstatus, welcher in der Klinik berechtigt, eigenständig Entscheidungen zu treffen, scheint eine Entscheidungsfindung auch präklinisch leichter zu fallen. Zwar weisen unsere Ergebnisse darauf hin, dass eine höhere Entscheidungskompetenz in Bezug auf palliativmedizinische Fragestellungen in der Präklinik existiert, aufgrund der kleinen Kohorte ist eine kausale Schlussfolgerung jedoch nicht möglich. Ferner sind das persönliche Engagement und Fortbildungen zur Thematik unberücksichtigt. Letztlich bleibt offen, ob die Berufserfahrung allein einen Einfluss auf die Sicherheit im Umgang mit palliativmedizinischen Patienten hat. Im Rahmen der Befragung wurden mittels PCEP-GR und BPW validierte Tools eingesetzt, um den Umgang mit Palliativpatienten zu ermitteln. Die Notärzte, unabhängig von einer palliativmedizinischen Weiterbildung, sind jedoch über ihre Berufserfahrung und eigene Vorstellungen in der Lage sich an die kultur- und kontextspezifischen Situationen anzupassen. Beide Aspekte sind unabdingbar für die Behandlung der Patienten und den Umgang mit Dritten. Hier haben die Palliativmediziner aufgrund ihrer Weiterbildung sicherlich einen Vorteil, da Situationen und Settings in deren Arbeitsalltag weitaus häufiger auftreten und eine größere Sicherheit im Umgang besteht.

Wissen über Palliativmedizin und Fragestellungen im Rettungsdienst sind und bleiben ein besonderes Tätigkeitsfeld und können nicht bei jedem Notarzt vorausgesetzt werden. Inwieweit Notärzte dort Unterstützung benötigen und sich wünschen, ist abhängig von deren Berufserfahrung, Interesse an entsprechender Thematik, eigenen Vorstellungen über Tod und Sterben sowie der Selbstwahrnehmung. Daraus lässt sich jedoch ableiten, dass Aus‑, Fort- und Weiterbildung in palliativmedizinischen Fragestellungen eine Optimierung der Versorgung von Palliativpatienten ermöglichen könnte. Alternativ wäre hier eine Unterstützung durch Palliativmediziner im Sinne der Telemedizin als Tool für die Kollegen und/oder die Etablierung eines standardisierten Algorithmus eine Option. Weiterhin wäre die Nutzung z. B. der „Mainzer Checkliste“ oder einer Patienten-Anweisung für lebenserhaltende Maßnahmen für Patienten in einer palliativen Situation („PALMA“; [32]), ergänzend zur ausführlichen Patientenverfügung oder der Palliativ-Ampel der Deutschen Palliativstiftung [33], die auf einer Seite die wichtigsten Informationen zu den gewünschten Maßnahmen aufzeigen, wünschenswert. Hierzu sollte darauf hingewirkt werden, dass Patienten in einer palliativen Erkrankungssituation ebendiese Informationen vorhalten und entsprechende Dokumente mit ihren Behandlern gemeinsam ausfüllen und somit den eigenen Willen dokumentieren. Hierdurch kann eine Übertherapie im konkreten Fall verhindert werden. Diese Checklisten können neben Rücksprache mit dem Hausarzt, anderen Behandlern, Telemedizin und SAPV-Teams eine sinnvolle Unterstützung sein. In einigen Landkreisen werden bereits Telemedizinprojekte zu verschiedenen Fragestellungen erfolgreich durchgeführt [18–20]. Patienten mit Anbindung an eine spezialisierte ambulante palliativmedizinische Versorgung (SAPV) haben die Möglichkeit einer telefonischen Kontaktaufnahme mit dem jeweiligen Team. Auch hier könnte eine bessere Vernetzung zwischen den einzelnen Beteiligten in der Versorgung von Palliativpatienten hilfreich sein, in dem zum Beispiel das SAPV-Team für Rückfragen des Notarztes im Sinne eines interkollegialen Austauschs zur Verfügung steht, um unnötige stationäre Einweisungen oder Therapien zu vermeiden.

Konfliktwahrnehmung zwischen Leben retten und Sterbebegleitung existiert in der Notfallmedizin, wie Salomon et al. bereits aufzeigen konnten. Das Problem, dass ein Konflikt aufgrund des Anspruchsdenkens vieler Notärzte und der Aufgabe eine Lebensgefahr abzuwenden entsteht, ist durchaus real. Die prognostische Frage spielt eine zentrale Rolle, welche aufgrund fehlender Informationen und dem Zeitdruck in der Notfallsituation oftmals unklar ist. Salomon und Kollegen schlussfolgerten, dass sich Entscheidungskonflikte nur durch fachliche Kompetenz vermindern lassen. Dazu sei dringend weitere notfallmedizinische Forschung und Weiterbildung vonnöten [11]. Allerdings haben Marung et al. bereits 2013 aufgezeigt, dass die bisherige 80-stündige Notarztausbildung keine ausreichenden Fähig- und Fertigkeiten für die Therapie von Patienten in palliativer Situation vermittelt [16]. Daran hat sich seitdem nichts geändert. Bei einer Zunahme an palliativmedizinischen Fragestellungen ist die Erweiterung der notärztlichen Ausbildung um dieses Themengebiet sinnvoll und wünschenswert. Überdies sollten die juristischen Aspekte und Fallstricke durch einen Juristen darlegt werden. Hieraus könnte somit eine höhere Rechtssicherheit im Umgang mit Patientenverfügungen oder dem mutmaßlichen Willen resultieren. In unserer Befragung gab ein Großteil der befragten Notärzte an, dass neben der vorgelegten Patientenverfügung auch der Wunsch der Angehörigen einen Einfluss auf die präklinische Therapie hat. Dies wurde von der Mehrheit als Belastung empfunden, wenn der Behandlungswunsch des Patienten unklar war.

Retrospektiv konnten Wiese et al. 2007 zeigen, dass Konflikte zwischen Notfallmedizin und Sterbebegleitung vorkommen. Insbesondere bei beruflich und palliativmedizinisch unerfahreneren Notärzten kam es (trotz Vorliegen einer palliativen Situation) häufiger zur stationären Einweisung von eben palliativen Notfallpatienten. Die allgemeine Unsicherheit über das korrekte Vorgehen bei Vorliegen einer palliativen Situation sowie der Zeitfaktor im Einsatz wurden als zentrale Einflüsse auf die Entscheidungsfindung identifiziert. Die Kombination aus Unsicherheit und Zeitdruck führte bei sechs von neun Patienten dazu, dass die jeweilige Situation in der durchgeführten Erhebung in einer stationären Krankenhauseinweisung mündete, obwohl die Patienten in einer vorliegenden Patientenverfügung ausdrücklich keine stationäre Behandlung wünschten [10]. Wiese et al. verdeutlichten, dass insbesondere mit zunehmender notärztlicher und palliativmedizinischer Erfahrung weniger Patienten in einem Finalstadium einer stationären Behandlung zugeführt werden. Des Weiteren wurden durch erfahrenere Notärzte, nach Feststellung der Prognose und des Vorerkrankungsstatus, weniger Reanimationen begonnen bzw. früher beendet. Insgesamt zeigen die Berufserfahreneren einen deutlichen Trend zu einer ambulanten und patientenorientierteren Behandlung, die ohne eine nachfolgende stationäre Versorgung endet [10].

Unsere Erhebung bestätigt dies. Die Erfahrung und die persönliche Sicherheit im Umgang mit Sterbenden wurden mit dem PCEP-GR-Fragebogen nach Schulz et al. überprüft. In unserer Kohorte zeigte sich eine mittlere positive Korrelation. Das bedeutet, dass eine längere Berufserfahrung mit einer besseren Vorbereitung auf den Umgang mit Sterbenden assoziiert ist. Die Berücksichtigung der ethischen Prinzipien nach Beauchamp und Childress, z. B. Respekt vor der Autonomie, sollten immer angewandt werden [21, 22]. Um mehr Sicherheit bei zeitkritischen Einsätzen zu erlangen, sind Aus‑, Fort- und Weiterbildungen hilfreich. Diese sollten verpflichtend angeboten werden, denn Patienten haben ein Recht auf eine adäquate Behandlung ohne Missachtung ihrer eigenen Wünsche [34].

Im aktuellen Konsensuspapier von 2023 stellen Michels und Kollegen heraus, dass zwar eine erweiterte S3-Leitlinie für Patienten mit einer nichtheilbaren Krebserkrankung existiert, es jedoch bisher keine palliativmedizinische Leitlinie für nichtonkologisch Erkrankte bzw. für Notfallpatienten in der Notaufnahme oder auf Intensivstationen gibt [6].

Im Rahmen des Medizinstudiums wurde der Querschnittsbereich Palliativmedizin (QB13) als Pflichtfach 2009 eingeführt [25]. Dies wurde in der Approbationsordnung entsprechend angepasst. Alle Notärzte, welche vor 2009 studiert haben, haben demzufolge keine systematische Ausbildung in der Palliativmedizin erhalten. Dies könnte einen Einfluss auf die Versorgung der Patienten haben oder gehabt haben. Und auch in der Diskussion um die Aus- und Weiterbildung von Notärzten nehmen Wiese und Kollegen Bezug auf die aktuelle Musterweiterbildungsordnung für die „Zusatzbezeichnung Notfallmedizin“ [12], welche keine Kenntnisse oder Fragestellungen im Bereich der Notfallversorgung von Tumorpatienten im finalen Krankheitsstadium einfordert [10]. Die Musterweiterbildungsordnung setzt für die Zusatzbezeichnung „Palliativmedizin“ [12] hingegen voraus, dass Kenntnisse zur Symptomkontrolle, der Gesprächsführung mit Patienten in der Finalphase, der Arbeit im multiprofessionellen Team sowie dem Umgang mit Therapieeinschränkungen, Sterbebegleitung und Vorausverfügungen vorhanden sind. Die Verknüpfung der Ausbildungsinhalte beider Musterweiterbildungsordnungen wäre zukunftsorientiert im Sinne einer notfallmedizinischen, bedürfnisorientierten und adäquaten Versorgung von Menschen am Ende des Lebens oder in schwierigen Erkrankungssituationen [10]. Wiese et al. sowie Marung et al. leiteten daraus ab, dass palliativmedizinische Fragestellungen in der notärztlichen Ausbildung berücksichtigt werden sollten [10, 16]. Als das Mindestmaß identifizierten sie die akute Symptomkontrolle bei Dyspnoe, eine adäquate Tumorschmerztherapie und die Behandlung akut bedrohlicher Symptome bei Tumorblutungen sowie die Kenntnisse über die Rechtsgrundlage von Patientenverfügungen.

In unserer Studie gab ein Großteil der befragten Notärzte an, dass Palliativmedizin fester Bestandteil der Aus- und Weiterbildung sein sollte. Zudem bestand bei den Befragten ein generelles Interesse zum Thema Palliativmedizin.

Wiese et al. schlussfolgerten, dass Palliativ- und Notfallmedizin keine Gegensätze, sondern sich gegenseitig ergänzende medizinische Bereiche sind [10]. In unserer Erhebung bestätigten die Befragten das Ergebnis. Daraus ist abzuleiten, dass palliativmedizinische Inhalte in die aktuelle notärztliche Ausbildung stärker integriert werden sollten. Ein Großteil der Teilnehmenden wünscht sich zudem mehr Fortbildungsinhalte zum Thema Palliativmedizin in der Notfallmedizin, worauf die Träger des Rettungsdienstes eingehen und entsprechende Fortbildungsthemen für ihr Personal anbieten sollten. Denn speziell solange noch keine flächendeckende ambulante Palliativversorgung besteht, werden Notärzte und Rettungsdienst weiterhin eine wichtige Rolle bei der Notfallversorgung von Palliativpatienten übernehmen müssen [10].

Im Jahr 2022 waren insgesamt ca. 5,7 Mio. Vorsorgeverfügungen im zentralen Vorsorgeregister der Bundesnotarkammer Deutschlands registriert. Davon waren 77 % mit einer Patientenverfügung kombiniert [9]. Roessler und Eulitz zeigten 2018, dass die Nachfrage nach einer Patientenverfügung bei der präklinischen Versorgung durch den Notarzt‑/Rettungsdienst bei den Behandelnden zu einer „Aussichtslosigkeitsannahme“ führen kann, bzw. bei An- und Zugehörigen Missverständnisse entstehen, dass grundsätzlich keine Maßnahmen mehr getroffen werden sollen und sodann auch beim Notarzt eine „negative therapeutische Grundeinstellung“ entsteht. Weiter bestätigte die Studie, dass eine palliativmedizinische Situation in der Präklinik medizinische und psychosoziale Herausforderungen für den gerufenen ärztlichen Kollegen vor Ort darstellen [4].

Lang und Wagner fanden 2007 heraus, dass vor allem Menschen eine Patientenverfügung ablehnen, die gesundheitlich unbelastet sind und in ihrem sozialen Umfeld weder mit Tod noch mit Sterben konfrontiert werden. Eine mangelnde Akzeptanz gegenüber Patientenverfügungen sowie ein Mangel an persönlicher Kenntnis über die letzte Lebensphase könnten ursächlich dafür sein, dass der Themenkomplex Sterben und Tod nicht thematisiert oder oftmals auch tabuisiert wird [8]. Notärzte sind insgesamt gehäuft mit dem Thema Tod und Sterben konfrontiert. Dadurch ist bei diesen im Vergleich zur Gesamtbevölkerung (28 %) auch wesentlich häufiger eine eigene Patientenverfügung vorhanden (65,7 %; [17]).

Langer et al. stellten 2016 fest, dass zwar durch das Inkrafttreten des „Patientenverfügungsgesetzes“ im Jahr 2009 die Patientenverfügung als Vorsorgeinstrument gesetzlich gestärkt wurde, dennoch existiert noch immer eine gesellschaftliche und fachliche Auseinandersetzung sowie Diskussion um ihre Vor- und Nachteile. Aber auch die Umsetzung der Patientenverfügung, dem vorausverfügten individuellen Willen eines einwilligungsfähigen Menschen zum Zeitpunkt der Erstellung, wird kontrovers diskutiert [7]. Die Alarmierung des Rettungsdienstes wird als eine Art mutmaßliche Einwilligung in notfallmedizinische Handlungen gesehen. Infolgedessen wird das Eruieren des Patientenwillens häufig vernachlässigt [10]. Eine vorhandene Patientenverfügung ist eine schriftliche Willensäußerung eines Patienten, in welcher der Betreffende über die durchzuführenden oder zu unterlassenden medizinischen Maßnahmen im Falle einer bestehenden Einwilligungsunfähigkeit entschieden hat. Diese kann die Entlastung von Dritten bedeuten. In einem emotionalen Ausnahmezustand, wie z. B. einem palliativmedizinischen Setting, ist mittels vorhandener Patientenverfügung die Entscheidung durch den Patienten selbst vorab getroffen und somit seine Patientenautonomie beachtet. Bei Fehlen einer Patientenverfügung werden in solch einer Situation die Entscheidungen von Ärzten im Einvernehmen mit den An- und/oder Zugehörigen, rechtlichen Vertretern, Betreuern oder Vorsorgebevollmächtigten entschieden. Hierbei ist dann die Patientenautonomie nicht mehr gewährleistet, was einen Einfluss auf die Integrität der körperlichen Unversehrtheit nach dem Grundgesetz Artikel 2, Absatz 2, Satz 1 und einer möglicherweise damit verbundenen Übertherapie darstellt. Auch ohne eine (wirksame) Patientenverfügung ist der Behandler stets verpflichtet, den mutmaßlichen Patientenwillen zu ermitteln. In einer präklinischen Notfallsituation ist einerseits der Zeitfaktor als auch häufig das Fehlen aller Befunde und Vorinformationen als wichtige Einflussfaktoren zu nennen. Selbst bei einer vorliegenden und auf die Situation zutreffenden Verfügung ist der Umgang und die Interpretation offenbar schwierig. Die ärztlichen Kollegen scheinen nicht ausreichend über zivil- und strafrechtliche Folgen bei Verstoß und/oder Missachtung der Patientenverfügung aufgeklärt zu sein. Kein Arzt darf einen Patienten gegen seinen vorausverfügten Willen behandeln. In der Literatur zeigen Studien von Padberg et al. und in der Schmitten et al., dass die Anwendbarkeit einer vorliegenden Patientenverfügung schwierig scheint [26, 27]. Eine der möglichen Sorgen könnten juristische Folgen bei Unterlassen von Therapien sein. Bei Missachtung einer vorliegenden Patientenverfügung drohen jedoch ebenso Konsequenzen, zu denen neben den zivil- und strafrechtlichen Aspekten (Körperverletzung) auch standesrechtliche Sanktionen gehören. Hierbei zeigt sich ein Dilemma, resultierend aus Nichtwissen über Konsequenzen bei Missachtung der Patientenverfügung und einem Gewähren der Patientenwünsche. Taupitz schreibt dazu: „An der Grenze von Leben und Tod gibt es keine Rechtssicherheit … Insgesamt bleibt die Errichtung wie auch die Befolgung einer Patientenverfügung eine höchst unsichere Sache. Das liegt vor allem daran, dass das Grundproblem der Auslegung von antizipativen Willenserklärungen vom Gesetz nicht gelöst werden konnte. In jedem konkreten Einzelfall muss die schwierige Frage beantwortet werden: Was hat der Betroffene für welche Situation wie verbindlich gewollt?“ [31].

Patientenverfügungen sind ein sensibles und intimes Thema, was eine mögliche Ursache für die geringe Anzahl an Studien zu diesem Thema sein könnte. Demzufolge ist es schwierig, unsere Arbeit in einen Kontext zu setzen. Eine aus 2016 stammende Studie zeigt, dass die Datenlage nationaler empirischer Studien zu dem Umgang mit Patientenverfügungen und zu ärztlichen Handlungsweisen am Lebensende bisher sehr gering ist [7]. Eine Untersuchung des Meinungsforschungsinstitutes Allensbach (Institut für Demoskopie Allensbach, IfD) aus dem Jahr 2014 kommt zu dem Ergebnis, dass 28 % der deutschen Bevölkerung eine Patientenverfügung besitzen. In der Altersgruppe > 60 Jahre beläuft sich der Anteil auf 51 % [17]. Präzisere Angaben zu Patientenverfügungen in Deutschland fehlen, da eine offizielle Registrierung in das Zentrale Vorsorgeregister der Bundesnotarkammer zwar möglich, aber nicht verpflichtend ist [8]. In unserer Befragung zeigte sich der Anteil der Teilnehmer mit vorhandener Patientenverfügung bei 65,7 %. Hier ist die Bedeutung zu den eigenen Wertevorstellungen und der Versorgung am Lebensende mit dem vorhandenen Wissen darüber sicherlich ein möglicher Einflussfaktor.

Ein Beitrag, um die Anwendungsprobleme von Patientenverfügungen und die Handhabe auf die medizinische Behandlung zu stärken, stellt das Advance Care Planning (ACP) dar. ACP ist als Prozess zu verstehen, welcher Erwachsenen in jedem Lebensalter, unabhängig vom Vorliegen oder Art und Stadium einer Erkrankung ermöglicht, die eigenen Werte, Lebensziele und Vorlieben bezüglich einer künftigen medizinischen Behandlung und Pflege mit ihren An- und Zugehörigen aber auch Ärzten, Therapeuten und Pflegenden zu teilen [28]. Gespräche über die eigenen Wünsche und die entsprechende Fixierung in schriftlicher Form mittels Patientenverfügung bilden demnach eine gute Möglichkeit, um Übertherapie am Lebensende zu vermeiden [29, 30]. Für den Notfall haben sich Zusammenfassungen der Patientenverfügung, wie z. B. die Palliativ-Ampel, bewährt. Auf diesen Übersichtsdokumenten werden die Kernpunkte der Patientenverfügung zusammengefasst, so dass der Notfallmediziner auf einen Blick erkennt, welche Maßnahmen vom Patienten gewünscht oder abgelehnt werden.

## Limitationen

Ein großer Anteil an Fachärzten mit Zusatzweiterbildungen in den Bereichen Intensiv- und Palliativmedizin zeigt eine hohe intrinsische Motivation der teilnehmenden Kollegen und könnte unsere Ergebnisse beeinflusst haben. Hierdurch kann ein Bias entstehen, in dem vermehrt Notärzte an der Befragung teilgenommen haben, die auch ein Interesse an der Studienfrage haben. Dadurch können die Ergebnisse überdurchschnittlich positiv ausfallen. Da es sich um eine freiwillige Befragung handelt, ist die Rückläuferquote gering, was die Repräsentativität der Ergebnisse beeinflussen könnte. Zudem waren neun Kollegen mit der Zusatzweiterbildung Palliativmedizin im Notarztdienst tätig und an der Befragung beteiligt, was einen möglichen Bias darstellt. Darüber hinaus ergeben sich gegebenenfalls auch regionale Unterschiede in der täglichen Praxis der präklinischen Versorgung, da es in Deutschland bereits Rettungsdienstbereiche geben mag, die eine gute palliativmedizinische Vernetzung mit anderen Leistungserbringern (z. B. SAPV-Diensten) anbieten. In unserer Stichprobe haben wir nur zwei Standorte befragen können, möglicherweise ist hier ein regionaler Einflussfaktor vorhanden. Unsere Kohorte ist zu klein, um zu einer Verallgemeinerung zu kommen.

Die Rechtsgültigkeit der Patientenverfügung wurde erst mit dem Patientenverfügungsgesetz 2009 bekräftigt. Bei allen Diskussionen und Studien vor 2009 besteht somit eine juristische Grauzone, die erst ab 2009 mit dem Inkrafttreten und der Rechtsverbindlichkeit geschlossen werden konnte [34]. Dieser Aspekt muss in der Interpretation und dem Vergleich mit Studien vor 2009 berücksichtigt werden.

## Zusammenfassung

Die Versorgung von Patienten mit onkologischen als auch nichtonkologisch lebenslimitierenden Erkrankungen wird in Zukunft weiter ansteigen und somit auch die Häufigkeit der Kontakte mit dem Rettungsdienst und Notärzten zunehmen. Eine unzureichende Vorbereitung auf den Umgang mit Palliativpatienten zeigt sich insbesondere bei Berufsanfängern. Hier gilt es ausreichende Angebote zu schaffen, um eine gute, patientenorientierte und sinnhafte Therapie in der Akutsituation zu leisten. Etwaige Algorithmen, Fortbildungskonzepte, Vernetzungen mit SAPV-Teams, Nutzung von etablierten Checklisten sowie Telemedizin könnten die Lücke hierbei schließen. Die Indikationen für die Inanspruchnahme des Rettungsdienstes sind mannigfaltig und bedürfen einer guten Vorbereitung, welche sich in der Anpassung der Musterweiterbildungsordnung widerspiegeln könnte. Weiterhin bestehen Unsicherheiten im Umgang mit Patientenverfügungen, die aber häufig nur von Menschen mit chronischen oder onkologischen Erkrankungen verfasst werden. Patientenverfügungen und deren Inhalte sind aus medizinischer, ethischer und juristischer Sicht zu bewerten. Im Rahmen von Notfallsituationen besteht hierbei aufgrund von Zeitdruck und wenigen Informationen ein Konfliktpotenzial. Aus Sorge vor juristischen Konsequenzen kann es zu einer Übertherapie am Lebensende kommen, wenn Palliativpatienten nichtsinnhafte Therapien angeboten werden und diese in einer Klinikeinweisung enden.

## Fazit für die Praxis

- Die Aus‑, Fort- und Weiterbildung von Notärzten in Palliativmedizin ist essenziell für die Versorgung der Patienten und wird von den Befragten für sinnvoll erachtet.
- Die Musterweiterbildungsordnung sollte hierauf eingehen und Inhalte für eine bessere Vorbereitung der Notärzte einfordern.
- Eine palliativmedizinische Versorgung wird speziell bei fehlenden ambulanten palliativmedizinischen Strukturen ein Bestandteil des Notarztdienstes bleiben.
- Konzepte wie Algorithmen, Vernetzung bekannter Strukturen wie Notarztdienst und SAPV sowie Telemedizin könnten die Versorgung optimieren und ergänzen.
- Sicherheit im Umgang mit Patientenverfügungen und Patientenwünschen am Lebensende würden die Versorgungsqualität und -zufriedenheit erhöhen.

## Data Availability

Die in dieser Studie erhobenen Datensätze können auf begründete Anfrage beim Korrespondenzautor angefordert werden.

## References

[1] Tiesmeier J, Salomon F, Lehning B, Tomaszczyk M, Henzler D, Jakob T (2017) Palliativmedizinisches Denken und Handeln im Notarztdienst – eine Befragung zur Strukturqualität. Notarzt 33(5):212–219. 10.1055/s-0043-118474

[2] von dem Knesebeck O, Vonneilich N (2016) In: Salomon F (Hrsg) Praxisbuch Ethik in der Notfallmedizin: Orientierungshilfen für kritische Entscheidungen. Medizinisch Wissenschaftliche Verlagsgesellschaft, Berlin

[3] Ruppert DB (2010) Palliativmedizin und Notfallmedizin: Zwei unterschiedliche medizinische Bereiche – Kenntnisse und Wünsche notfallmedizinischen Personals. Georg-August-Universität zu Göttingen. 10.53846/goediss-870

[4] Roessler M, Eulitz N (2018) Notarzt und Palliativmedizin. 10.19224/ai2018.430 29564473

[5] Wiese C, Graf B, Hanekop G Notärztliche Versorgung von Palliativpatienten. Dtsch Med Wochenschr 133(41):2078–2083 10.1055/s-0028-1091246 18985559

[6] Michels G, John S, Janssens U, Raake P, Schütt KA, Bauersachs J et al (2023) Palliativmedizinische Aspekte in der klinischen Akut- und Notfallmedizin sowie Intensivmedizin: Konsensuspapier der DGIIN, DGK, DGP, DGHO, DGfN, DGNI, DGG, DGAI, DGINA und DG Palliativmedizin. Med Klin Intensivmed Notfmed 10.1007/s00063-023-01016-9 PMC10244869 37285027

[7] Langer S, Stengel I, Fleischer S, Stuttmann R, Berg A Umgang mit Patientenverfügungen in Deutschland. Dtsch Med Wochenschr 141(09):e73–e79 10.1055/s-0042-104038 27123734

[8] Lang F, Wagner G Patientenverfügungen in Deutschland: Bedingungen für ihre Verbreitung und Gründe der Ablehnung. Dtsch Med Wochenschr 132(48):2558–2562 10.1055/s-2007-993097 18033650

[9] Wurm S, Spuling SM, Reinhard AK, Ehrlich U (2023) Verbreitung von Patientenverfügungen bei älteren Erwachsenen in Deutschland 10.25646/11568 (verfügbar unter: https://edoc.rki.de/handle/176904/11290)

[10] Wiese C, Bartels U, Ruppert D, Quintel M, Graf BM, Hanekop GG Notärztliche Betreuung von Tumorpatienten in der finalen Krankheitsphase. Anaesthesist 56(2):133–140. 10.1007/s00101-006-1129-x 17216503

[11] Adams HA, Salomon F Entscheidungskonflikte am Notfallort. Anästhesiol Intensivmed Notfallmed Schmerzther 35(5):319–325. 10.1055/s-2000-320 10858842

[12] Bundesärztekammer (2018) Musterweiterbildungsordnung für Notfallmedizin und Palliativmedizin (S 400–402, 409–411)

[13] Rupp et al (2024) Resuscitation (un-)wanted: Does anyone care? A retrospective real data analysis. Resuscitation 198:110189. 10.1016/j.resuscitation.2024.110189 38522733

[14] Tiesmeier J et al (2018) Palliativmedizinische Notfallpatienten Teil 1 – Handlungsoptionen und Symptomkontrolle. Retten 7:25–36. 10.1055/s-0043-119201

[15] Leitlinienprogramm Onkologie (Deutsche Krebsgesellschaft, Deutsche Krebshilfe, AWMF) (2020) Palliativmedizin für Patienten mit einer nicht-heilbaren Krebserkrankung, Langversion 2.2. https://www.leitlinienprogramm-onkologie.de/leitlinien/palliativmedizin/. Zugegriffen: 11. Aug. 2024 (AWMF-Registernummer: 128/001OL)

[16] Marung H et al (2013) Der Notarzt beim Palliativpatienten: Evaluation eines neuen Kurskonzepts. Notarzt. 10.1055/s-0033-1349584

[17] IfD Allensbach (2014) Deutlicher Anstieg bei Patientenverfügungen. www.ifd-allensbach.de/studien-und-berichte/allensbacher-kurzberichte.html. Zugegriffen: 29. Okt. 2024 (Allensbach Kurzbericht)

[18] Skorning M et al Stellenwert und Potenzial der Telemedizin im Rettungsdienst. Notfall Rettungsmed. 10.1007/s10049-010-1397-5

[19] Brokmann JC et al (2017) Telemedizin: Potenziale in der Notfallmedizin. Anästhesiol Intensivmed Notfallmed Schmerzther 52(02):107–117. 10.1055/s-0042-108713 28222471

[20] Humburg D et al (2022) Der Telenotarzt als „Einsatzleiter“ zur Stabilisierung eines kritisch kranken Patienten. Notfall Rettungsmed 25(8):578–584. 10.1007/s10049-021-00945-2 PMC8459140 34580575

[21] Marckmann G (2000) S 499–502

[22] Beauchamp, Childress (2009) Principles of biomedical ethics

[23] van Oorschot B, Lipp V, Tietze A, Nickel N, Simon A (2005) Einstellungen zur Sterbehilfe und zu Patientenverfügungen: Ergebnisse einer Befragung von 727 Ärzten. Dtsch Med Wochenschr 130(6):261–265. 10.1055/s-2005-83741 15692898

[24] Weber M, Müller M, Ewald H (2005) Onkologe 11:384–391. 10.1007/s00761-005-0852-2

[25] Böhm L et al (2023) Anaesthesiologie 72(12):863–870 37994928 10.1007/s00101-023-01356-3 PMC10692016

[26] in der Schmitten J, Rixen S, Marckmann G (2011) Patientenverfügungen im Rettungsdienst (Teil 1). Notfall Rettungsmed 14:448–458. 10.1007/s10049-011-1527-8

[27] Padberg J, Esser A, Lomberg L et al (2014) Ethische Probleme im Umgang mit Reanimation und Patientenverfügung in der Notaufnahme. Notfall Rettungsmed 17:500–506. 10.1007/s10049-014-1909

[28] Sudore RL et al (2017) Defining advance care planning for adults: a consensus definition from a multidisciplinary Delphi panel. J Pain Symptom Manage 53(5):821–832.e1. 10.1016/j.jpainsymman.2016.12.331 (Epub 2017 Jan 3, PMID: 28062339, PMCID: PMC5728651) 28062339 PMC5728651

[29] Wright AA, Zhang B, Ray A et al (2008) Associations between end-of-life discussions, patient mental health, medical care near death, and caregiver bereavement adjustment. JAMA 300(14):1665–1673. 10.1001/jama.300.14.1665 18840840 PMC2853806

[30] Heyland DK, Ilan R, Jiang X et al (2016) The prevalence of medical error related to end-of-life communication in Canadian hospitals: results of a multicentre observational study. BMJ Qual Saf 25(9):671–679. 10.1136/bmjqs-2015-004567 26554026

[31] Taupitz J (2010) Das Patientenverfügungsgesetz: Mehr Rechtssicherheit? In: Sturma D, Heinrichs B, Honnefelder L (Hrsg) Jahrbuch für Wissenschaft und Ethik, Bd. 15. De Gruyter, Berlin, Boston, S 155

[32] Gerth M et al (2009) Brauchen wir eine spezielle Notfall-Patientenverfügung? – Ergebnisse einer Pilotbefragung. Notarzt 25:189–193

[33] https://www.palliativstiftung.com/de/shop/gedrucktes/vorsorgemappe

[34] Katzenmeier C (2002) Arzthaftung, S 57

[35] Bundesjustizministerium Patientenautonomie am Lebensende. Ethische, rechtliche und medizinische Aspekte zur Bewertung von Patientenverfügungen. Bericht der Arbeitsgruppe „Patientenautonomie am Lebensende“ vom 10. Juni 2004. www.bmj.bund.de/files/-/695/Bericht_AG_Patientenautonomie.pdf

